# Health Effects Related to Wind Turbine Noise Exposure: A Systematic Review

**DOI:** 10.1371/journal.pone.0114183

**Published:** 2014-12-04

**Authors:** Jesper Hvass Schmidt, Mads Klokker

**Affiliations:** 1 Institute of Clinical Research, University of Southern Denmark, Odense, Denmark; 2 Department of Audiology, Odense University Hospital, Odense, Denmark; 3 Department of ENT Head and Neck Surgery, Odense University Hospital, Odense, Denmark; 4 Department of ENT Head and Neck Surgery & Audiology, Copenhagen University Hospital, Copenhagen, Denmark; 5 Faculty of Health and Medical Sciences, Copenhagen University, Copenhagen, Denmark; Queensland University of Technology, Australia

## Abstract

**Background:**

Wind turbine noise exposure and suspected health-related effects thereof have attracted substantial attention. Various symptoms such as sleep-related problems, headache, tinnitus and vertigo have been described by subjects suspected of having been exposed to wind turbine noise.

**Objective:**

This review was conducted systematically with the purpose of identifying any reported associations between wind turbine noise exposure and suspected health-related effects.

**Data Sources:**

A search of the scientific literature concerning the health-related effects of wind turbine noise was conducted on PubMed, Web of Science, Google Scholar and various other Internet sources.

**Study Eligibility Criteria:**

All studies investigating suspected health-related outcomes associated with wind turbine noise exposure were included.

**Results:**

Wind turbines emit noise, including low-frequency noise, which decreases incrementally with increases in distance from the wind turbines. Likewise, evidence of a dose-response relationship between wind turbine noise linked to noise annoyance, sleep disturbance and possibly even psychological distress was present in the literature. Currently, there is no further existing statistically-significant evidence indicating any association between wind turbine noise exposure and tinnitus, hearing loss, vertigo or headache.

**Limitations:**

Selection bias and information bias of differing magnitudes were found to be present in all current studies investigating wind turbine noise exposure and adverse health effects. Only articles published in English, German or Scandinavian languages were reviewed.

**Conclusions:**

Exposure to wind turbines does seem to increase the risk of annoyance and self-reported sleep disturbance in a dose-response relationship. There appears, though, to be a tolerable level of around L_Aeq_ of 35 dB. Of the many other claimed health effects of wind turbine noise exposure reported in the literature, however, no conclusive evidence could be found. Future studies should focus on investigations aimed at objectively demonstrating whether or not measureable health-related outcomes can be proven to fluctuate depending on exposure to wind turbines.

## Introduction

In recent years suspected health-related effects of exposure to wind turbine noise have attracted much public attention. Whether or not this exposure can result in an array of described symptoms and disorders has been widely debated. It has been reported that noise from wind turbines can lead to such symptoms as dizziness, nausea, the sensation of ear pressure, tinnitus, hearing loss, sleeping disorders, headache and other symptoms. Additionally, the term “Wind Turbine Syndrome” has been coined to describe the association of these symptoms to wind turbine noise exposure [1]–[6]. However, the level of scientific evidence in wind turbine research, evaluated by several comprehensive reviews, is poor, as most of the research used to reach the conclusions found in these studies has been based on mere case reports and other similar studies [7]–[11]. It has also been argued that most of the symptoms supposedly related to wind turbine noise exposure could be psychosomatic ones stemming from a fear of wind turbines rather than any real adverse health effects [12]. Furthermore, reports in the scientific literature which have tried to establish a causal relationship between wind turbine noise and adverse health effects have tended to initiate heated debates between the authors and their readers, with critics often claiming that there was an insufficient amount of high quality evidence of a direct-dose response relationship between the noise exposure and the symptoms [9], [13], [14].

In order to shed light on the question of causation, researches frequently seek a statistically significant dose-response relationship. Statistically significant relationships between exposure and symptoms may not be shown to be causal without knowing if there is a dose-response relationship. The aim of the present study is to systematically analyse the literature and conclude if there is any evidence to support these theories of adverse health effects caused by exposure to wind turbines.

### Guidelines, Recommendations and Requirements for wind turbine noise

Noise from wind turbines is generated to a lesser degree by the rotory hub; however, virtually all other wind turbine noise is generated by the downward movement of the rotating blades which result in the characteristic audible swishing pulses [15]–[17]. During the night these swishing pulses can become more dominant, and pulses from several wind turbines in the same vicinity can propagate in phase and lead to increased pulse sounds with increased sound pressure levels of 5 dB [18]. This amplitude modulation of the sound can also become more prominent under certain meteorological conditions [19]. Furthermore, noise from wind turbines will increase with any increase in the ambient wind speed [20], [21]. This amplitude modulating sound is often considered to be the most annoying aspect of wind turbine noise, and this has led to suggestions of incorporating the level of amplitude modulation as a measurement parameter for setting regulations for these noise measurements [22]–[24].

Noise is often measured as A-weighted equivalent sound pressure levels (L_Aeq_) during a certain period of time. To then calculate L_den_, 10 dB is added to the A-weighted equivalent sound pressure levels (L_Aeq_) during the night and 5 dB is added to these noise levels during evening periods. If L_Aeq_ is constant throughout the day and night, L_den_ can be calculated by the addition of 6.4 dB to the measured L_Aeq_ [25]. L_den_ is measured at a height of 10 meters, and it is dependent on the wind speed, the landscape and the turbine type [25]. In several countries wind turbine noise has been limited to a maximum allowable level of L_Aeq_ at 35–44 dB, depending on the given wind speed and the special noise sensitivity in areas with low levels of background noise [9], [21], [26]–[28]. In Denmark, for example, the maximum level - L_max_, corresponding to a L_Aeq_ of 42–44 dB or 37–39 dB in noise sensitive areas, is dependent on the wind speed (8 or 6 m/s respectively) [29]. In general noise levels in residential areas are calculated from noise prediction models; however, these noise prediction models have often been found to over-predict wind turbine noise levels at the point of the receivers [30].

Infrasound is considered to be sound of frequencies below 20 Hz, and low-frequency sound is considered to be sound between 20–200 Hz. Infrasound originates from many different sources in the environment including compressors, ventilation and traffic noise [31]. It has been demonstrated that wind turbines can cause low-frequency sound exposure of above 20 dB in the homes of close neighbours [32]. Most countries do not have regulations regarding infrasound and low-frequency noise from wind turbines, with the exception of Denmark where low-frequency sound in the 10–160 Hz range is limited to an A-weighted level (L_pALF_) of 20 dB [29].

## Methods

The supporting PRISMA checklist is available as supporting information; See Checklist S1.

The objective of the present study was to analyze the literature systematically, and to determine if there was any statistical evidence of adverse health effects from exposure to wind turbine noises. The literature reviewed here included literature from both peer-reviewed scientific sources as well as internet sources which were not necessarily peer-reviewed. All types of studies investigating any relationship between wind turbine noise exposure and health-effect outcomes were included in the systematic review. Furthermore, with the purpose of aiding in the analysis and interpretation of the findings of the systematic review, a separate review of issues related to wind turbine exposure was also conducted. Focus was given in particular to finding additional technical information with respect to the size and character of wind turbine noise, as well as information regarding documented community opinions of wind turbines.

**Figure 1:**
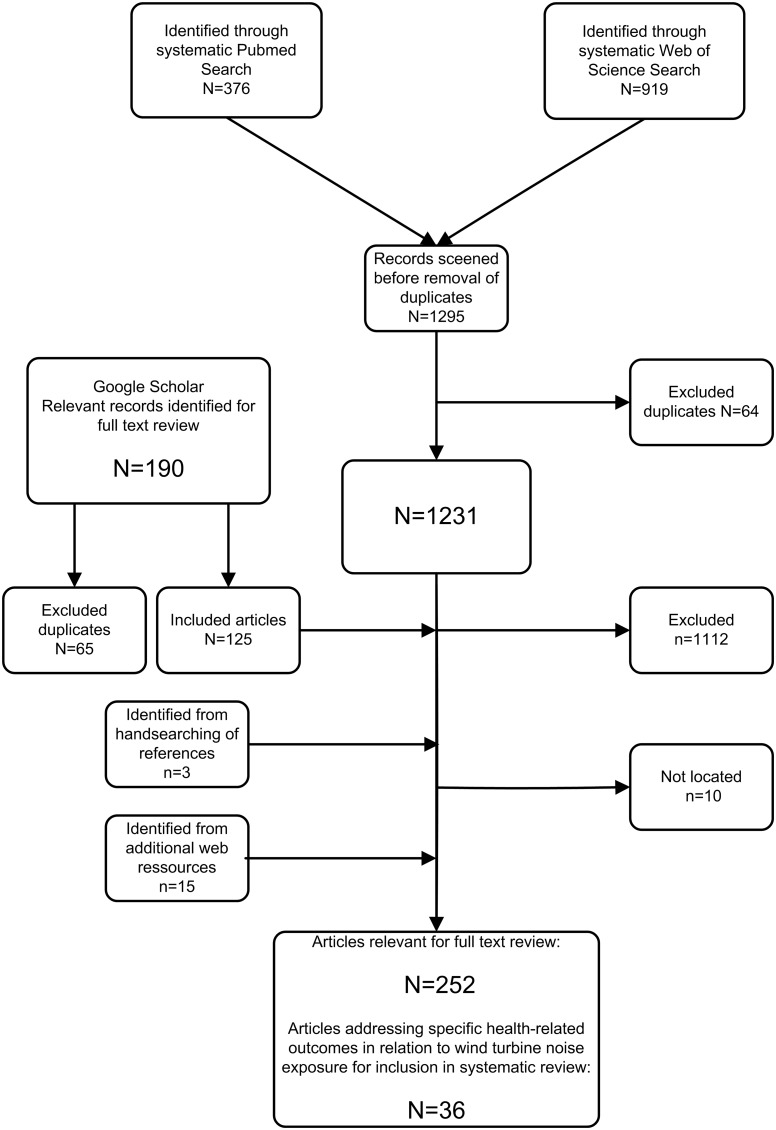
Search strategy for relevant publications.

A PubMed search was conducted using the search string: wind turbines OR wind turbine OR wind farm OR wind farms. Additionally, a Web of Science search was conducted using the search string: (wind turbines OR wind turbine OR wind farm OR wind farms) AND (health OR noise OR annoyance OR tinnitus OR vertigo OR epilepsy OR headache) (Figure 1). Both database searches were performed again for a final time on the 9^th^ of June 2014, and included all relevant reports published up until that time. No limits in language were used in the database searches.

Duplicates of articles were removed, and the titles and abstracts of all records were screened. Articles were then selected for full-text review, dependent upon whether the content of the article concerned wind turbines and related health effects on humans. Of the articles selected for full-text review, reported health effects included noise annoyance and psychological aspects related to the opinions of communities regarding wind turbine noise, as well as specific studies exploring noise exposure from wind turbines. Only articles in English, German and Scandinavian languages were selected for full-text review. Articles containing specific environmental issues and problems related to biology and wild life, as well as more technical articles regarding wind turbine mechanics, were not selected for full-text review.

Additionally, Google Scholar (http://scholar.google.dk/) was searched with the same search string previously used for the Web of Science search (See Figure 1). Articles from all years were retrieved in the initial search, while all patents and citations were excluded. (Google Scholar is a search engine that shows the 1000 most relevant web-resources). Several additional searches were also performed with limitations set to articles from 2014, 2013–2014 and 2010–2014. Based on these searches, publications were selected for full-text review based on the same criteria as described above. The final search was performed on the 9^th^ of June 2014. Following this selection procedure, duplicates of previously retrieved articles were removed.

Google Scholar may not necessarily retrieve all relevant sources of, in particular, non-peer reviewed sources, and it was also evident from searches in Google Scholar that several additional websites contained a large number of relevant publications. Therefore, publications listed at the following websites: https://www.wind-watch.org/, http://www.windturbinesyndrome.com/and http://waubrafoundation.org.au/were also searched and screened by using the same selection procedure as described above. Publications were only retrieved for full-text review, however, if they were not already retrieved in the previous searches with PubMed, Web of Science and Google Scholar.

The reference lists of selected publications were also searched for additional articles pertaining to health-related issues believed to be related to wind turbine noise.

All articles investigating the association between wind turbine noise exposure and any suspected health-related outcomes were then identified from among the articles selected for full-text review, and these identified articles were included in the systematic review. Originally the systematic review had only attempted to investigate and, if possible, provide evidence for any association between wind turbine noise exposure and health-related outcomes such as noise annoyance, sleep disturbance, any kind of psychological distress including mental and concentration problems, tinnitus, vertigo, headache and epilepsy, as these symptoms had been reported in case reports as resulting from wind turbine exposure. The data extraction was, however, not limited to these variables. Other health-related outcomes could be included in the review as well, should these variables later be identified as important during the review process. Thus, information regarding any evidence of health-related effects associated with wind turbine noise exposure was extracted from the included articles by one reviewer and confirmed by the second reviewer. Disagreements were resolved by discussion of the selected articles. Duplicate publications from the same study population were included in the study to present additional evidence for health-related effects related to wind turbine noise, if this evidence was not previously reported in other publications. It was also specified when several publications reported data from identical study populations. Extracted information from different studies addressing similar health-related outcomes related to wind turbine exposure were summarized in tables where study differences in terms of study populations and the exposure assessment were described. It was stated when a specific association between wind turbine noise exposure and a health-related outcome was found to exist.

No limiting criteria regarding the quality of the research was used initially in the selection process of the articles for the systematic review of health-related effects in relation to wind turbine noise exposure. Any potential risk of bias identified in the selected studies was assessed afterwards and reported specifically as a part of the quality assessment of the included studies in the systematic review.

## Results

Literature searches from PubMed and the Web of Science identified 1231 publications after the removal of duplicates (Figure 1). Only articles related to wind turbines and health-related effects were selected for further full text review, and, for this reason, 1112 publications were excluded.

In total 119 publications from the Web of Science and PubMed databases and additional 125 publications identified from Google Scholar were selected for further evaluation (Figure 1) after excluding duplicates. Fifteen publications were added from Internet sources regarding wind turbines. Additionally, three publications were included after reviewing the reference lists of the selected publications, and 10 of the selected publications could not be retrieved. Thus, a total of 252 unique publications were included in the full-text review. Thirty-five publications investigating health-related outcomes of exposure to wind turbines were identified from the systematic literature search to be included in the systematic review [1], [33]–[66]. In addition, one article that calculated the expected annoyance of sound exposure to wind turbines based on previous results from Janssen et al. 2011, was also included [33], [67]. Thus, 36 publications fulfilled the inclusion criteria and were included in the systematic review. Two publications by Janssen et al. (2009, 2010), two by Pedersen et al. (2006, 2008), and one publication by Nissenbaum et al. (2011) were published in conference proceedings. The results in these publications were identical to the meta-analysis published by Janssen et al. 2011, and to the studies from Sweden and the Netherlands published by Pedersen et al. (2007, 2009) and to the study from the U.S.A. published by Nissenbaum et al. (2012). Thus, only Janssen et al. 2011, Pedersen et al. 2007 and 2009 and Nissenbaum et al. 2012 were used in the systematic review [33], [37], [39], [40], [58]–[62]. Likewise, only Pawlaczyk-Luszczynska et al. 2014 was used for this review since Pawlaczyk-Luszczynska et al. 2013 reported identical results in a conference proceeding [46], [63]. As such, 30 publications, after the exclusion of the aforementioned six conference publications, were identified as specifically investigating health-related outcomes of exposure to wind turbine noise.

Four of these 30 publications were identified as case series [1], [64]–[66]. Case series studies report adverse health effects which are hypothesized to be a result of exposure to wind turbines. Case studies in general may be affected by selection and information bias which may also be true for the selected case studies in this review. This means, that these case studies may be biased and, as such, contribute fairly week evidence towards forming any conclusions about causation. The studies can, however, be hypothesis-generating in terms of a causal relationship.

The remaining 26 publications that investigated a relationship between exposure to wind turbine noise and adverse health effects were cross-sectional studies. These studies used a stratified approach where subjects with low or no exposure were compared to subjects with high exposure to wind turbine noise [36]–[56], [67]. One of these studies with a limited sample size (11 exposed, 10 unexposed) used longitudinal health data related to wind turbine noise exposure [57]. Three of these studies were meta-analyses of previous cross-sectional studies [33]–[35]. With such cross-sectional studies it is thereby possible to assess a dose-response relationship between exposure to wind turbine noise and adverse health effects. Selection bias and information bias, however, will likely occur. Cross-sectional studies can, therefore, not be used to determine any specific causal relationships.

Thus, the evidence presented in this systematic review had to rely on case-series reports and cross-sectional studies. Meta-analyses could increase the sample size, but the level of evidence was still dependent on the original cross-sectional studies included in the meta-analysis.

None of the included studies investigated the relationship of health effects and exposure to low-frequency noise or infrasound; however, infrasound and low-frequency noise emission from wind turbines were measured and studied in a number of the publications retrieved from the 252 articles initially selected [2], [32], [68]–[77].

### Health effects related to infrasound and low-frequency sound exposure from wind turbines

#### Infrasound

While no study conducted so far has examined the potential adverse health effects related to specific infrasound exposure, this subject has been widely debated as a possible explanation for suspected health effects of wind turbine noise exposure even when the infrasound is not audible [1], [37], [42], [45], [46], [52], [53], [66]. Infrasound in general may be audible at high sound pressure levels; however, infrasound from wind turbines is subaudible unless one is very close to the wind turbine rotor [68], [72], [78]. Wind turbine infrasound levels for frequencies of up to 20 Hz were measured between 122–128 dB near wind turbines using G-weighting as recommended for the measurement of infrasound [32]. At further distances, however, between 85–360 meters from other wind turbines, G-weighted sound pressure levels were measured between 61–75 dB [32], [69]. In addition, measurements taken from large wind turbines above 2 MW at distances ranging between 68–1000 meters gave an infrasound exposure of between 59–107 dB(G), as summarized in a review by Jakobsen [70]. Smaller wind turbines below 2 MW measured at 80–500 meters distance were recorded as giving an infrasound exposure of 56–84 dB(G) [70]. Similar infrasound exposures were measured at 350 meters from a gas-fired power station (74 dB(G)), at 70 meters from major roads (76 dB(G)), at 25 meters from the waterline at the beach (75 dB(G)) as well as at 8 kilometres inland from the coast (57 dB(G)) [69]. Even when the infrasound exposure from wind turbines is not audible outdoors, infrasound in the 5–8 Hz range can still lead to a rattling of doors and windows which is audible indoors and can be an annoyance to those living in close proximity to wind turbines [73].

Wind turbines do emit infrasound, but it remains unknown if exposure to infrasound from wind turbines can lead to adverse health effects. It has also been hypothesised that infrasound may contribute to the amplitude-modulated nature of wind turbine noise which can then contribute to the perception of this noise [79], [80].

Some physiological changes have, however, been demonstrated in humans exposed to infrasound as shown in one functional MRI study where 110 dB infrasound at a 12 Hz tone activated areas of the primary auditory cortex in the brain [81]. Infrasound at 6 Hz and 130 dB was also able to affect Distortion Product Otoacustic Emissions (DPOAE) in humans [82]. The exposure in these studies was above 100 dB(G) and may be audible to some individuals. Of further note, it has been demonstrated in a double-blinded study that patients with Meniere’s disease experience significant relief or even curative effects by using a Meniett pressure device which applies pressure of sinusoidal pulses of 6 Hz [83].

Some evidence suggests that even inaudible sound may affect the delicate structure of the ear and the vestibular organ. A recent review of several animal studies demonstrated that small physiological changes could be detected in the cochlear outer hair cells when these animals were exposed to infrasound. The outer hair cells of the cochlea were more sensitive to infrasound compared to the inner hair cells [84]. There exists as yet no human data comparable to that of these animal studies, so it is therefore still unclear if such theoretical affections of the inner ear structures can explain why some individuals have symptoms like tinnitus, vertigo and Meniere’s disease [4].

Exposure to inaudible infrasound from wind turbines has also led to speculations that adverse health effects resulting from this exposure are perhaps psychological in nature [12]. In two recent randomized and controlled psychological experiments 54 and 60 subjects respectively were randomized into groups with either positive or negative expectations towards wind turbine noise and then informed separately about either the potential benefits or the supposed harmful effects and symptoms related to wind turbines and infrasound exposure. The subjects were shown either positive or negative videos about wind turbines and related health effects prior to the experiments. These studies demonstrated that the subjects randomized to the groups with negative expectations reported significantly more symptoms both when exposed to infrasound (p<0.01) and to sham infrasound (no sound) (p<0.01), as well as after exposure to audible wind turbine noise compared to the baseline (p<0.001) [85], [86]. Thus, these experiments support the hypothesis that a subset of the population conditioned to dislike wind turbines may be more sensitive to adverse effects after infrasound exposure itself or wind turbine noise in general [85], [86]. It should be noted that discrete sound exposure periods in a listening room may not be comparable to wind farm noise; however, positive or negative expectations towards wind turbine noise or any other noise would seem to affect self-reported health outcomes. Such psychological expectations may influence the opinion of a subset of the population who will then fear the potential health effects of wind turbines [8]. Furthermore, there can be a general resistance in the population towards a nearby planned location of wind turbines close to residential areas. This phenomenon has been termed “Not In My Back Yard (NIMBY)”, and it relates to the resistance often seen when a wind farm project or any other project (e.g. airports, highways, chemical plants) is planned near a residential area, regardless of whether or not that project is actually harmful or just perceived to be so [87], [88].

Thus, it remains unknown if exposure to infrasound from wind turbines does cause adverse health effects or if these potential health effects are the results of psychological mechanisms. Moreover, no studies so far have specifically examined the relationship between G-weighted sound pressure levels of infrasound with wind turbine noise exposure and health effects, and, likewise, no studies have demonstrated an influence of infrasound on specific vestibular diseases.

#### Low-frequencies

Wind turbines have been shown to produce a relatively large amount of noise in the low-frequency spectrum [32], [89]. Wind turbine low-frequency noise can be more intense compared to other well-known sources of low-frequency noise such as road traffic noise and aircraft noise [89]. Furthermore, the low-frequency noise can increase with an increase in turbine size [32]. In fact, this noise is not particularly different when compared to other known sources of low-frequency noise from road traffic noise and industry [29].

Sound pressure levels of nine wind turbines (2.3–3.6 MW) were measured in Denmark, and the distances which equalled L_Aeq_ of 35 dB were calculated. The distances were found be between 629–1227 meters from the rotor of the wind turbine. At this distance the level of the infrasound was 54–59 dB(G) and the low-frequency noise was between 26.7–29.1 dB(A). The highest octave band was found to be 250 Hz, and this means that low frequencies play an important role regarding the noise measured in neighbouring areas of wind turbines. Half of the measured room/wind turbine combinations actually demonstrated that the low-frequency limit of 20 dB set by Danish legislation was exceeded [32]. Furthermore, noise generated by wind turbines can lead to ground vibrations [68], [71]. These ground vibrations are, however, small since walking or running 50 meters from the measurement point, elicited larger outdoor vibrations than a wind turbine located 90 meters away [68]. However, the perception of sound and sensation of airborne vibrations from i.e. wind turbines has been demonstrated to be higher indoor compared to outdoor and the vibrations indoor were detected as recurrent low-frequency pulses which are likely to be more annoying compared to a more constant noise [2], [71], [90].

Vibrations from low-frequency sounds are reported to be the cause of vibro-acoustic disease (VAD) [91]. VAD is reported to happen when long-time exposure to low-frequency sounds occurs [92], [93]. However, VAD has not yet generally been accepted as a clinical disease by the medical community as reviewed by Chapman and St George [91].

### Relationship between noise annoyance and sound exposure

Noise annoyance is not directly studied as a primary outcome in most of the case studies; however, it is evident from these studies that many subjects complain about noise from wind turbines [1], [64], [66].

Several reasons can explain why wind turbine noise probably causes more annoyance than other sound sources. Wind turbines are often placed in areas where background noise levels are low. People living in these areas may have sought out tranquillity and have likely accustomed themselves to the silence, which may influence their annoyance level regarding unwanted sounds in their environment [5], [94]. Furthermore, any changes in their surroundings or their environment will probably introduce a level of annoyance in the case of at least some individuals [5], [8].

As shown in Table 1, which summarizes studies on annoyance and wind turbine noise, a dose- response effect of noise exposure and noise annoyance has been demonstrated. Two studies (754+351 subjects) were conducted in Sweden, one study in the Netherlands (725 subjects), one study in Poland (156 subjects) and one study in Japan (747 subjects) with different questionnaires assessing noise annoyance used than those in the aforementioned studies from Sweden, the Netherlands and Poland [38]–[40], [46]. All five studies demonstrated a significant relationship between A-weighted sound exposure and wind turbines and annoyance [38]–[40], [44], [46]. All studies were cross-sectional studies, and they used a questionnaire-based survey which was combined with either direct sound measurements or estimated sound emission levels outside the subjects’ dwellings [38]–[40], [44], [46]. All studies asked for subjective answers regarding the degree of annoyance towards different sound sources to mask the true purpose of the questionnaire [38]–[40], [44], [46]. In general, the selection of geographical areas in which to conduct these studies was quite large, encompassing several different areas, thus helping to limit selection bias. In the study from Japan, for example, a control group of 332 subjects not exposed to wind turbines was also included for comparison. While wind turbine noise was found to be the most annoying sound source in the exposed group, traffic noise was perceived as the most annoying sound in the control group [43].

**Table 1:** Relation between annoyance and sound exposure to wind turbines.

Studies	N	Dose- response-relationship	Effects	Other factorsinfluencing annoyance
Jansen et al. [33] 2011(meta analysis ofPedersen et al.2004,2007,2009[38]–[40].	1820	Yes	Highly exposed subjectsmore annoyed comparedto less exposed subjects.	Noise sensitive subjects (↑)Visiblewind turbines (↑)Age (↑)Economicbenefits (↓)
Pedersen 2011 [35]. (Asubpopulation of samestudy populations asJansen et al. 2011 [33]).	1755	Yes	Highly exposed subjectsmore annoyed comparedto less exposed subjects.	Economic benefits (↓) – analyseswere adjusted for economic benefits,but only in analyses with data from Pedersen et al. 2009.
Pedersen and Larsman2008 [34] (meta-analysisof Pedersen et al. 2004 and 2007 [38], [39].	1095	Yes	Highly exposed subjectsmore annoyed comparedto less exposed subjects.Effect was independenton terrain.	Negative evaluation of wind turbines(↑)Visual attitude towards windturbines for subjects who could seethe wind turbines and to a lowerdegree for subjects who could notsee the wind turbines (↑)Increasedvertical visual angel is correlated towind turbine noise and annoyance (↑)
Pedersen et al.2009Bakker et al.2012 [36], [40], [41].	725	Yes	Highly exposed subjectsmore annoyed comparedto less exposed subjects.	Noise sensitive subjects (↑)Visiblewind turbines (↑)Economic benefit(↓)Build-up area opposed to ruralarea without main road (↑)Ruralarea with main road (↓)
Pedersen et al. 2004[38], [41], [47].	341	Yes	Highly exposed subjectsmore annoyed comparedto less exposed subjects.	Noise sensitive subjects (↑)Negativeattitude to visible wind turbines(↑)Negative attitude to windturbines in general (↑)
Pedersen et al 2007[39], [41], [47].	754	Yes	Highly exposed subjectsmore annoyed comparedto less exposed subjects.	Noise sensitive subjects (↑)Attitudeto visible wind turbines (↑)Attitudeto wind turbines in general (↑)
Pawlaczyk-Luszczynska et al.2014 [46].	156	Yes	Highly exposed subjectsmore annoyed comparedto less exposed subjects.	Noise sensitive subjects (↑)Attitudeto visible wind turbines (↑)Attitude to wind turbines in general(↑)Sensitivity to landscape littering(↑)Negative self-assessment ofphysical health (↑)Wind turbineswere found to be the most annoying sound source.
Aslund et al. 2013 [67].Based on calculationsfrom Pedersen et al.2009 and Bakker et al. 2012 andJansen et al. 2011[33], [36], [40].	8123 theoretically exposedsubjects.522 areparticipatingreceptors.	Yes (Dose-response relationship derived from other studies).	Highly exposed subjects close to wind turbines calculated to be more frequently annoyed and very annoyed.	Participating residents in wind farmprojects (↑)Annoyance outdoorcalculated to be higher thanannoyance indoor.
Shepherd et al.2011 [42].	39 subjects.158controls.	Not related to sound – related to distance.	Annoyance not directlycompared between subjects and controls.	Annoyance decreased perceivedgeneral health as well as physical,social and environmental qualityof life scores for the control grouponly. Subjects reported, however,lower environmental quality of life scores compared to controls.
Kuwano et al. 2013 [43].	747 subjects.332 controls.	Not related to sound.	Proportion of annoyed subjectshigher in wind turbine exposed subjects	All kinds of noise sourcesincreased annoyance in bothgroups. Subjects in the windturbine group found wind turbinesas the most annoying sound source.
Yano et al. 2013 [44].	747 subjects.	Yes	Highly exposed subjects moreannoyed compared to less exposed subjects.	No difference in dose-responsecurves between cold and warmareas. Living near the sea (↓).(Waves may mask wind turbine sounds). Noise sensitivity (↑)Landscapedisturbing (↑)Environmental interest (↑)
Morris 2012 [50], [51].	93 households.	Not related to sound.	56% of households are annoyedduring night time within 0–5 km. from the wind turbinescompared to 40% ofhouseholds living within 0–10 km from wind turbines.	No influencing factorswere investigated.
Schafer 2013 [54].	23 households.	Not related to sound.	66% of subjects affected bynoise at night.	No influencing factorswere investigated.
Schneider 2012 [55], [56].	23 households, 25 household in follow-up.	Not related to sound.	85.7%/(87.7% in follow-up study) were disturbed from daytime noise. 100% fromnight time noise in follow-up.	No influencing factorswere investigated.
Thorne 2012 [52].	25	Not related to sound, but sound levels measured.	91% were annoyed indoor.	No influencing factorsexcept living near wind turbines wereinvestigated.

Additionally four studies ranging from 23 to 93 households were conducted near four different specific wind farms in Australia (Table 1) [50]–[52], [54]–[56]. These studies reported that 40 to 91% of households were annoyed. Response rates between 23 to 40% were reported in only two of the studies [50], [51], [55], [56].

The studies from Sweden and the Netherlands were used in a meta-analysis where L_den_ was calculated from the measured L_Aeq_ reported in the original studies [33]. To calculate L_den_ an average correction factor of 4.7 dB was used as earlier suggested by van den Berg (2008) to account for differences in wind conditions and different terrains in the different studies. By calculation of L_den_ this study could compare the degree of annoyance in relation to L_den_ and this value could be compared to other well-known sources of environmental noise such as road traffic noise and noise from airports. The meta-analysis showed that noise from wind turbines was perceived as more annoying compared to noise from road traffic, airports and trains at similar values of L_den_ [33]. Age, general noise sensitivity and visual disturbance by wind turbines were positively associated with annoyance whereas economic benefit was significantly negatively associated with annoyance. The data from the two Swedish studies were also combined in an additional analysis and it was demonstrated that noise annoyance from wind turbines was significantly correlated to swishing, whistling, resounding and pulsating sounds from wind turbines [34]. Furthermore, a model for the dose-response relationship between sound exposure and the risk of high annoyance due to sound exposure to wind turbines was established [33]. The degree of annoyance has in general been reported to be between 10–45% of the population if the sound exposure was above 40 dB(A) but less than 10% of the population will be annoyed if the sound exposure is below 35 dB(A) [38]–[40]. In a planned wind farm project where the noise exposure was calculated based on the results from the meta-analysis by Janssen et al., 17 to 18% of the 8123 recipients living within a distance of 1 km from the wind turbines were expected to be rather or very annoyed when outdoors [33], [67]. On the other hand, it was demonstrated in a field study from the United States that the degree of annoyance was only 4% in a population living within a distance of approximately 600 meters to wind turbines [95].

Experimentally it has been shown that wind turbine noise does not differ substantially from traffic noise when the wind turbine noise is not known of in advance [96]. However, wind turbine noise is poorly masked by road traffic noise unless the exposure to wind turbine noise is at an intermediate level (35–40 dB(A)) [97], [98]. Wind turbine noise has distinctive features which allow for detecting that type of noise from amongst other sound sources at low signal-to-noise ratios. This means that focussing on the sound can increase noise annoyance [96]. It has been shown that wind turbine noise can be masked with natural background noise. In order to mask the sound completely the background noise needs to exceed the noise from the wind turbines with 8–12 dB [99]. An increase of background noise with 8–12 dB is not practical, but the perceived loudness of noise and annoyance from wind turbines is reduced if the background noise is at the same level or higher than the wind turbine noise [99], [100].

It was calculated that 330 dwellings in the Netherlands were exposed to wind turbine noise exceeding L_den_ by as much as 50 dB and that 440.000 inhabitants were exposed to L_den_ above 29 dB. Of these 440.000 inhabitants, 1500 were expected to be severely annoyed [89]. The estimation of this noise exposure at different dwellings may, however, have been altered by atmospheric changes, so it was further calculated that the sound exposure could be up to 5 dB lower and 10 dB higher than predicted under neutral conditions. It is generally believed that noise limits for wind turbines should be set at a level where fewer than 10% of exposed people are annoyed. A limit of 45 dB in the Netherlands has been estimated to annoy 5.2% of the exposed inhabitants [89].

### Relation between wind turbine noise exposure and sleep disturbance

Table 2 summarizes studies investigating the relationship between noise exposure to wind turbines and sleep disturbance. Reports from case studies indicated that many subjects living near wind turbines complained of sleep disturbance [1], [64]–[66]. These results were supported by the finding of a dose-response relationship between self-reported sleep disturbance and A-weighted noise exposure in three out of four larger epidemiological studies from Sweden, the Netherlands and Poland [35], [36], [46]. Furthermore, a disturbed sleep was also found to be higher among exposed subjects compared to unexposed control subjects in three studies from Japan (754 subjects, 332 controls), the U.S.A. (38 subjects, 41 controls) and New Zealand (39 subjects, 158 controls) [37], [42], [43]. The Pittsburg Sleep Quality Index (PSQI) was used as an outcome measurement in the American study and in studies, from Australia (25 subjects) and Canada (396 subjects) [37], [45], [52]. The Australian study showed lower PSQI in the wind turbine group compared to known population values [52]. The studies from the U.S.A. and New Zealand both demonstrated a significant relationship between PSQI results and the distance to the wind turbines. Selection bias is a concern in these studies, however, as only a few selected wind farms were included in the studies, and the study from Canada had a response rate of only 8% [37], [42], [45]. Surveys of single wind farms in Australia, including 23–93 households within 0–10 km from the wind farms, investigated sleep disturbance along with the noise annoyance reported above. Twenty-nine to ninety-two percent of exposed households reported disturbed sleep in these studies (Table 2) [50]–[52], [54]–[56]. A larger survey from New Zealand (614 subjects) found only 42 subjects with disturbed sleep, but this study only investigated subjects living within 2–10 km to the wind farm. A study from Canada collected self-reported sleep disturbance complaints amongst other health-related outcomes. The data was collected from an Internet survey where subjects reported health data. This study found a significant relationship between the distance to wind turbines and undue tiredness (p<0.03). However, disturbed sleep (p<0.08) showed only a borderline significance in relation to the distance from the wind turbines [49].

**Table 2:** Relation between sound exposure to wind turbines and sleep disturbance.

Study	N	Dose- response-relationship	Effects	Other factors influencing sleep
Nissenbaum et. al. 2012 [37].	38 subjects near wind turbines.41 controls far from wind turbines.	Not related to sound but sleep scores related to distance.	Subjects near wind turbines had worse sleep (Pittsburg Sleep Quality Index and Epworth Sleepiness Scale score) compared to subjects far from wind turbines.	
Bakker et al. 2012 [36].	725	Yes	Highly exposed subjects reported more frequent sleep disturbances.	Sleep disturbance higher in urban areas where subjects were disturbed by traffic noises, people leaving the disco, animals.
Pedersen et al. 2011 [35].	1755	Yes/No	Highly exposed subjects reported more disturbed sleep in 2 out of 3 studies.	Pedersen et al. 2004 and 2009 did report an association between sound exposures and sleep disturbance. Pedersen et al. 2007 did not find an association.
Pawlaczyk-Luszczynska et al. 2014 [46].	156	Yes	Highly exposed subjects suffered significantly more of insomnia (p<0.05).	Negative self-assessment of physical health (↑) Wind turbines were found to be the most annoying sound source.
Kuwano et al. 2013 [43].	747 subjects.332 controls.	Not related to sound – related to distance.	Proportion of subjects with affected sleep was slightly higher in wind turbine exposed subjects.	All kinds of noise sources increased sleep disturbance in both groups. Subjects in the wind turbine group found wind turbines as the most disturbing sound source.
Shepherd et al. 2011 [42].	39 subjects.158 controls.	Not related to sound – related to distance.	Perceived sleep quality poorer in subjects (wind turbine exposed) compared to controls (not exposed).	Worse sleep with increased noise sensitivity in wind turbine exposed. General health, physical and psychosocial health increased with better perceived sleep quality.
Krogh et al. 2011 [49].	102 subjects with health problems.	Not related to sound.	Sleep disturbance more frequently reported, but not significantly (p = 0.08) different in subjects living close to wind turbines compared to subjects living further away.	Excessive tiredness was reported significantly increased (p = 0.03) in subjects living within 350–673 meters from wind turbines compared to subjects living between 700–2400 meters from wind turbines.
Lane 2013 [57].	11 exposed.10 unexposed.	Increased awakenings were related to sound levels above 45 dB(A).	Slightly but not significantly worse sleep parameters in the exposed group measured with actigraph.	Reasons of awakening were not related to wind turbine noise. Use of the bath-room by a child or partner were the most commonly reported sources of awakening. No correlation between distance to wind turbines and sleep efficiency were found. Overall uneven correlation between subjective and objective sleep parameters.
Paller 2014 [45].	396	Not related to sound but sleep scores related to distance.	Subjects near wind turbines had worse sleep (Pittsburg Sleep Quality Index) (p<0.01) compared to subjects far from wind turbines.	Analyses were controlled for age, gender and county.
Harry 2007 [66].	42	Not related to sound.	More than 70% of cases reported impaired sleep.	No control group. Cases are just reported to live near wind turbines.
Iser 2004 [65].	19	Not related to sound.	8/19 = 42% reported disturbed sleep.	No control group. Cases were just living near wind turbines.
Morris 2012 [50], [51].	93	Not related to sound.	39% of households had disturbed sleep within 0–5 km. from the wind turbines compared to 29% of households living within 0–10 km from wind turbines.	No influencing factors were investigated.
Wind Concerns Ontario [64].	112	Not related to sound.	48% reported sleep disturbance.	No influencing factors except living near wind turbines were investigated.
Schafer 2013 [54].	23 households.	Not related to sound.	51% of subjects affected by sleep disturbance.	No influencing factors except living near wind turbines were investigated.
Schneider 2012 [55], [56].	23 households. 25 households in follow-up.	Not related to sound.	78.5% had disturbed sleep. 100% had disturbed sleep in follow-up study.	No influencing factors except living near wind turbines were investigated.
Thorne 2012 [52].	25	Not related to sound, but sound levels measured.	92% noted a change in sleep patterns.	No influencing factors except living near wind turbines were investigated.
Pierpont 2009 [1].	38 subjects from 10 families.	Not related to sound.	86% reported disturbed sleep.	No influencing factors except living near wind turbines were investigated.
Phipps [53].	614 households.	Related to distance.	Disturbed sleep was reported by 42, frequently disturbed sleep by 21 and 5 were affected most of the time.	No influencing factors except living near wind turbines were investigated.

Whereas most studies collected only subjective information about sleep disturbance, some studies attempted to also collect objective longitudinal sleep data over several nights. By using an Actigraph, sleep was monitored and related to noise measurements in the sleeping room. The study had a limited sample size; however, and no difference in objective sleep quality in relation to the noise exposure was observed in the 11 subjects exposed to wind turbines compared to the 10 unexposed subjects.

Noise from various environmental factors can affect sleep if the noise is pronounced at night [101].

Noise annoyance, self-reported sleep disturbance and psychological stress were all related to increasing sound pressure levels of wind turbines [35], [36], [42]. The impact of wind turbine noise was stronger for people living in rural areas with less background noise from other environmental factors. Sleep disturbance was only seen at high exposure levels above 45 dB(A), and sleep disturbance was significantly related to annoyance [36]. It was not possible, however, to conclude that sleep disturbance was caused directly by wind turbine noise, as other environmental noise sources could have played a role as well [36]. On the other hand, noise annoyance was not significantly correlated to sleep disturbance within a distance of two kilometers from the wind turbines, as had been reported in the study from New Zealand. Sleep, and ones physical and environmental quality of life were, however, affected in the wind turbine exposed group as reported above, and the authors suggested that both sleep disturbance and noise annoyance could have caused the observed degradation of health-related quality of life in the wind turbine exposed group [42]. Sleep disturbance was only weakly associated to A-weighted sound pressure levels in the first Swedish study and in the Dutch study if in- and outdoor noise annoyance were also included in the models. This demonstrates a correlation between noise annoyance and sleep disturbance and that noise annoyance may be a mediator of sleep disturbance or that sleep disturbance may induce annoyance [35].

### Relation between wind turbine noise and other health parameters

Table 3 summarizes the findings from studies investigating the association between wind turbine noise and psychological distress. Psychological symptoms such as memory and concentration problems, anxiety and stress were frequently reported in case series of subjects exposed to wind turbine noise [1], [64]–[66]. Furthermore, noise annoyance was significantly associated to psychological distress [36]. Several studies measured the WHO-quality of life (WHOQOL) and found that physical health scores among wind turbine exposed subjects were lower than those of the unexposed controls as well as those of the general population (Table 3) [42], [48], [52]. The social and psycho-social scores in a study from New Zealand, however, did not differ between exposed and unexposed subjects in the initial investigation, and neither were these scores altered in a follow-up study two years later [42], [48]. Nonetheless, the general health of the turbine-exposed group was reported to be significantly lower when compared to controls [42], [48].

**Table 3:** Psychological distress.

Study	N	Dose- response-relationship	Effects	Other factors influencingpsychological distress
Bakker et al.2012 [36].	725	Yes	Highly exposed reportedpsychological distress(General health questionnaire).	Annoyance influencepsychological distressand in this casepsychological distressis not predicted bysound-exposure.
Nissenbaum et al.2012 [37].	38 subjects nearwind turbines41controls far from windturbines.	Not related tosound but sleepscores related todistance.	Subjects near wind turbines had worsemental scores (Mental ComponentScore of SF-36) compared tosubjects far from wind turbines.	
Shepherd et al.2011 [42].	39 subjects.158controls.	Not related tosound.	No differences found in psychologicaland social health-related quality of life(WHOQOL) questionnaire parameters.	
McBride et al.2013– a follow-upof Shepheardet al. 2011 [42], [48].	Selected from 56exposed housesand 250 controlhouses.	Not related tosound.	WHO-quality of life (WHOQOL) didnot change in the follow-up period inthe exposed group. The physical domainand general satisfaction with healthscored significantly lower in theexposed group compared to the controlgroup in the most recent study.	Amenity decreased significantly in the control group over time. Amenity was stable in the exposed group over time.
Harry 2007 [66].	42	Not related tosound.	More than 50% of cases reportedanxiety and stress.	No control group. Cases are just reported to live near wind turbines.
Iser 2004 [65].	19	Not related tosound.	8/19 = 42% reported stress and likelysymptoms.	No control group. Cases were just living near wind turbines.
Wind ConcernsOntario 2009[64].	112	Not related tosound.	A majority reported stress, anxiety,excessive tiredness, depression.	No influencing factors except living near wind turbines were investigated.
Thorne2012 [52].	25	Not related tosound, but soundlevels measured.	Mental component score of SF-36 weremuch lower than expected from knownpopulation scores.	No influencing factors except living near wind turbines were investigated.
Pierpont2009 [1].	38 subjects from10 families.	Not related tosound.	93% reported memory andconcentration problems.	No influencing factors except living near wind turbines were investigated.

Another general health questionnaire (SF-36) was used to measure mental and physical component scores in wind turbine noise exposed subjects [37], [52]. Mental component scores were significantly lower with decreasing distance between the dwelling and the wind turbines, and the scores were also lower if they were compared to those of the general population [37], [52]. These studies may have been affected by selection bias, and the two wind farms investigated in the study by Nissenbaum et al. do not seem to be comparable in terms of exposure. The sound was measured at various distances from the wind turbines and then compared. It is evident that the sound levels measured at various distances were quite different in the two wind turbine parks. It is not, however, known if weather conditions or different terrains were the main causes of these differences, and it is also difficult to determine if the wind turbines were responsible for the sleep disturbance and low mental component scores in this study [37].

Associations between A-weighted sound pressure levels and subjective tinnitus and diabetes were demonstrated in one of the previous Swedish studies by Pedersen et al. [35]. As pointed out by the authors this could be a coincidental finding due to a multiplicity of logistic regressions since this finding was only demonstrated in one out of three studies investigating the association between sound exposure and tinnitus or diabetes. No significant associations between A-weighted sound pressure levels and headache, impaired hearing, chronic disease, cardiovascular diseases, high blood pressure, undue tiredness, irritability, tension, or stress were observed [35].

Case series studies of wind turbine noise exposed subjects often report headache, vertigo, tinnitus and hearing loss as frequent symptoms [1], [64]–[66]. Likewise, 8 out of 23 households reported headache and 4 out of 23 households reported dizziness in a study from a single Australian wind farm [54]. Self-reported symptoms like tinnitus, hearing problems, headache, stress and anxiety were not shown to be significantly related to the actual distance from the wind turbines, although one study did approach statistical significance for the symptom of tinnitus in relation to the distance from the wind turbines (p<0.08) [45], [49]. Symptoms of self-reported vertigo (p<0.001) were also increased for residents living closer to wind turbines in this study [45].

It is hypothesized that sound may affect the vestibular organ in the inner ear even at subaudible levels [84], [102]. A clinical test of vestibular function such as the vestibular-evoked myogenic potential (VEMP) test demonstrates that the vestibular system is sensitive to acoustic frequencies. Some vestibular diseases are known to be sensitive to change in pressure, such as perilymphatic fistula (PLF), superior canal dehiscence (SCD) and Meniere Disease (MD). The SCD (known as “a third window”), a defect in the superior semi-circular canal can give rise to Tullio phenomenon with sound-induced dizziness [103]. Such pressure-sensitive vestibular patients, however, have not as yet been evaluated with regard to wind turbine noise exposure even though such speculations have been made [84]. In our own clinical experience we have never seen PLF, SCD or MD patients complaining of aggravation of vestibular symptoms due to neighbouring wind turbines.

It has been further speculated that rotating wind turbine wings passing through the sunlight can induce epileptic attacks in sensitive subjects because the sunlight will be seen to flicker on the horizon. This phenomenon is known in the field of aviation medicine and can actually disqualify a pilot at the aeromedical health check-up due to the risk assessment associated with flying a turbo prop plane or helicopter. If light flickers at a frequency around 3 Hz there is a known risk that this can induce an epileptic attack in sensitive subjects [104]. The risk has been calculated as minimal in the case of large wind turbines which are unlikely to rotate fast enough to create an abruption of sun-light of more than three times per second, but there could be a risk with smaller wind turbines [105]. Shadow flickering is, however, a concern. It is often described in case series reports and studies from single wind farms and it may contribute to the overall annoyance from wind turbine exposure [1], [50], [51], [53].

## Discussion

Noise from wind turbines results in significant annoyance for neighbours of wind turbines, and the level of annoyance is related to the A-weighted sound exposure [33]–[35], [38]–[40], [44], [46], [106]. It has been shown that the sound exposure from wind turbine noise increases noise annoyance by dose-responsive degrees, and this annoyance may be the primary mediating agent causing sleep disturbance and increased psychological distress [35], [36]. On the other hand, it is also possible that sleep disturbance may lead to increased annoyance. Self-reported sleep disturbance was found to be significantly related to the given sound exposure and more frequently reported from subjects living closer to wind turbines compared to subjects living further away [35]–[37], [42], [46], [49].

Annoyance was significantly related to psychological distress and the mental component scores of SF-36 were significantly affected in wind turbine exposed subjects in some studies [36], [37], [52]. However, no differences in the psychological and social health-related quality of life (WHOQOL) questionnaire parameters were observed in other studies [42], [48].

The quality of the studies included in this review is quite varied. There are five cross-sectional studies of reasonable sample size from which a dose-response relationship between sound exposure and health outcomes, particularly in relation to annoyance and sleep disturbance, was demonstrated [33]–[36], [38]–[41], [44], [46]. Selection bias and recall bias may, however, still have affected the outcomes of these studies, and it should be acknowledged that the sample groups in these studies were from many different wind turbine sites located in quite different geographical regions. Virtually all of the studies did point toward an association between wind turbine exposure and annoyance or sleep disturbance; however, one of the significant limitations of these cross-sectional studies is their inherent inability to evidence a clear causal relationship between exposure to wind turbines and health-related outcomes. It is therefore not known with certainty if the association between wind turbine exposure and health-related outcomes is caused by sound exposure, visual disturbance, economic aspects or something else. Cross-sectional studies are simply more explorative by nature.

Several studies investigated sleep disturbance and psychological distress in relation to an unexposed or low exposed control group [37], [42], [43], [48]. Sleep disturbance and psychological distress were only reported in self-reported questionnaires which increase the risk of introducing information bias into the study. Selection bias is a concern as well if the study population is not representative for an entire population of wind turbine exposed subjects. As such, selection bias as well as information bias related to the outcome are of concern and may potentially affect conclusions drawn by the studies. The study by Kuwano et al., however, was relatively large, investigating several different geographical areas of Japan. Thus selection bias would be less of concern in this study [43].

Several case reports have raised concerns that wind turbine noise may lead to various symptoms such as tinnitus, vertigo and headache. Until now, however, of these suspected symptoms, only tinnitus has been shown to have an association with A-weighted sound exposure, and that only in a single study out of three similar studies [35]. Neither was this association between wind turbine noise exposure and tinnitus supported in other studies either [45], [49]. These findings, as well as the finding of an association of A-weighted sound exposure to diabetes in one out of three similar studies, may be a result of multiple logistic regressions which can lead to spurious conclusions [35]. These results need to be confirmed by additional studies, before sufficient evidence can be established to support this association.

Most studies investigating a dose-response relationship between sound exposure and annoyance have used calculated values of L_Aeq_ or L_den_ based on model assumptions of sound propagation from wind turbines over distance [35], [38]–[40], [44], [46], [106]. It might be relevant to include another type of sound weighting rather than just the A-weighting in future studies. In fact G-weighted sound exposure was estimated in one study, but these values were not related to adverse health effects [46]. Furthermore, it has been demonstrated that other characteristics of the noise from wind turbines may correlate better with noise annoyance than the frequently used A-weighted metric [107], [108]. It seems evident that low-frequency sound exposure may increase with increasing turbine size [32]. However, others reports have demonstrated that the content of low-frequency sounds from wind turbines may not be particularly different compared to other environmental background noises [29]. Sound from several wind turbines may increase the sound pressure level of swishing pulses from the wind turbines, and this could be a factor relevant to the perceived noise annoyance [15], [20], [34], [71], [109]. It may therefore be relevant to focus future studies on serial monitoring of the sound exposure to include the nature of the amplitude-modulated sound and the low-frequency sound exposure in dwellings near wind turbines. It is known that wind turbine noise is quite dependent on the existing wind speed, and health-related effects of wind turbine noise could, therefore, be speculated to fluctuate depending on the different noise levels at different wind speeds [110]. It has also been suggested that G-weighed sound exposure levels could be used as well to demonstrate the exposure to infrasound [32]. An experimental study, however, found a possible link between the psychological expectations of symptoms following both actual infrasound and a sham sound exposure trial. In these trials a difference between the infrasound and sham sound could not be demonstrated [85]. These results should, however, be interpreted with caution, as laboratory conditions may not be comparable to the real life exposure of wind turbine noise.

One study has already measured objective sleep parameters in relation to sound exposure, but the sample size of the study was a limiting factor in reaching any conclusions [57]. Future studies should focus more on objective measurements of health-related disorders in relation to wind turbine noise exposure. Sleep could be monitored parallel with sound exposure measurements, and stress hormones could be measured as well. Objective measurements of health can be a valuable asset in combination with more subjective measurements when used in questionnaires regarding annoyance from wind turbine noise. Both types of data can be related to sound exposure measurements, and it could be relevant to report both A- and G-weighted sound exposure measurements as well as a thorough characterisation of exposure in the low-frequency area including the maximum peak values of the swishing pulses from wind turbines.

It is currently known that traffic noise exposure may increase the risk of cardiovascular disease and diabetes [111], [112]. The mechanism here could be increased stress and reduced quality of sleep which can increase the risk of cardiovascular diseases and diabetes [111], [112]. It is not yet known if wind turbine noise exposure during the night could result in identical health effects.

Furthermore, it should also be acknowledged that some patients might have symptoms of a functional somatic syndrome, describing persistent bodily complaints for which no objective findings supporting the symptoms can be found [113]. Many of the core symptoms of the wind turbine syndrome, such as tinnitus, headache, dizziness, nausea, sleep disorders and lack of concentration, as reported by subjects exposed to wind turbine noise, show a similar bodily distress as described in other functional somatic syndromes [1], [113]. Events like accidents and potential environmental health hazards can induce a functional somatic syndrome in certain individuals, and this may be potentiated by mass hysteria in the media [113], [114]. Issues of possible wind turbine health impacts have also been addressed by the mass media using emotionally-charged words and phrases such as “dread” and “poorly understood by science”, and fright tactics like these may well have contributed to a mass hysteria regarding wind turbines [115], [116]. There are, nonetheless, numerous reports of many complaints related to wind turbine noise from various case studies [1], [6], [51], [55], [66]. These symptoms could be stress-related, and it is possible that these symptoms could occur as a result of sleep disturbance. On the other hand, these symptoms could be psychosomatic and explained as another sort of a functional somatic syndrome [12].

## Conclusion

At present it seems reasonable to conclude that noise from wind turbines increases the risk of annoyance and disturbed sleep in exposed subjects in a dose-response relationship. There seems to be a tolerable limit of around L_Aeq_ of 35 dB. Logically, accepting higher limits in legislations may lead to increased numbers of annoyed subjects. It therefore seems reasonable to conclude that a cautious approach is needed when planning future wind farms. Furthermore, there is an indication that noise annoyance and sleep disturbance are related and that disturbed sleep potentially can lead to adverse health effects. These conclusions are, however, affected by a potential risk for selection and information bias even in the larger cross-sectional studies providing the current best evidence. The evidence for adverse health effects other than sleep disturbance is primarily supported by case-series reports which certainly may be affected by various sources of bias. Larger cross-sectional surveys have so far been unable to document a relationship between various symptoms such as tinnitus, hearing loss, vertigo, headache and exposure to wind turbine noise. One limitation causing this could be that most studies so far have only measured L_Aeq_ or L_den_. An additional focus on the measurement of low-frequency sound exposure as well as a more thorough characterisation of the amplitude modulated sound and the relationship between objective and subjective health parameters could lead to different conclusions in the future. Finally, in regards to the objective measurement of health-related disorders in relation to wind turbine noise, it would be valuable to demonstrate if such health-related outcomes fluctuate depending on exposure to wind turbine noise.

## Supporting Information

Checklist S1 **PRISMA 2009 checklist.**
(DOC)
